# Prefrontal damage in childhood and changes in the development of personality: a case report

**DOI:** 10.1590/S1980-57642013DN70100019

**Published:** 2013

**Authors:** Valéria Santoro Bahia, Leonel Tadao Takada, Leonardo Caixeta, Leandro Tavares Lucato, Claudia Sellitto Porto, Ricardo Nitrini

**Affiliations:** 1 MD, PhD, Behavioral and Cognitive Neurology Unit, Department of Neurology, Hospital das Clínicas, University of São Paulo School of Medicine, São Paulo SP, Brazil.; 2 MD, Behavioral and Cognitive Neurology Unit, Department of Neurology, Hospital das Clínicas, University of São Paulo School of Medicine, São Paulo SP, Brazil.; 3 MD, PhD, Associate Professor of Neuroscience, Federal University of Goiás, Goiânia GO, Brazil. Coordinator, Cognitive and Behavioral Neurology Unit, Hospital das Clínicas.; 4 MD, PhD, Neuroradiologist, Instituto de Radiologia do Hospital das Clínicas da Faculdade de Medicina da Universidade de São Paulo, São Paulo SP, Brazil. Centro de Diagnósticos Brasil, São Paulo SP, Brazil.; 5 PhD, Behavioral and Cognitive Neurology Unit, Department of Neurology, Hospital das Clínicas, University of São Paulo School of Medicine, São Paulo SP, Brazil.; 6 MD, PhD, Full Professor, Behavioral and Cognitive Neurology Unit, Department of Neurology, and CEREDIC, Hospital das Clínicas, University of São Paulo School of Medicine, HC/FMUSP.

**Keywords:** traumatic brain injury, neuropsychology, frontal lobe, social behavior

## Abstract

Frontal lobe lesions are associated with behavioral abnormalities and executive dysfunction. When these lesions occur early in life, the symptoms are even more severe as the anatomical and functional substrates underlying personality and behavior are damaged, distorting normal modulation by interaction with the psychosocial environment. We present a case of a 40-year-old man who suffered a frontal lobe lesion at the age of nine years and developed impulsivity, disinhibition and inappropriate behaviors while showing some preservation of insight. Brain MRI revealed lesions to bilateral orbitofrontal cortex, ventromedial prefrontal cortex, anterior cingulate gyri and genu of the *corpus callosum*, which were more extensive on the right side. The right prefrontal dorsolateral cortex was severely damaged, whereas the right ventrolateral prefrontal cortex was spared. We will discuss the correlation of the damaged pre frontal regions with the symptoms presented by the patient.

## Abstract (pt)

Lesões no lobo frontal são associadas com sintomas de distúrbios do comportamento e disfunção executiva. Quando a lesão ocorre em fase precoce da vida, os sintomas são ainda mais intensos pois o substrato anatômico e funcional da formação da personalidade e comportamento está danificado, então, a sua modulação decorrente da interação com o meio psicossocial será distorcida. Apresentamos aqui o caso de um homem de 40 anos que sofreu uma lesão no lobo frontal aos nove anos de idade, com sintomas de impulsividade com manutenção parcial da autocrítica, desinibição e comportamento inapropriado. A ressonância de crânio evidenciava lesões bilaterais do córtex orbitofrontal da porção anterior do giro do cíngulo e do joelho do corpo caloso e do córtex ventromedial, mais extensa à direita. O córtex pré-frontal dorsolateral estava extensamente acometido à direita, enquanto o córtex pré-frontal ventrolateral parecia poupado. Discutiremos a correlação das áreas pré frontais lesadas com a sintomatologia do paciente.

## INTRODUCTION

The relationship between frontal lobe lesions and personality changes has been highlighted since the paradigmatic description of Phineas Gage's case (apud Macmillan).^1^ Another source of evidence includes prefrontal lesions that occur during childhood, a period in which the personality is being formed. In comparison to such lesions occurring during adulthood, personality changes caused by these prefrontal lesions in children tend to be more severe, with consequences that become apparent during development.^2^.

## CASE REPORT

The history was reported by the patient and her mother. The patient was a 40-year-old civil servant first seen in 1994. At that time, he reported having punched his adolescent son in front of his friends for little reason. After the event, he had suicidal thoughts. He also mentioned having attacked his friends for trivial reasons and that he refrained from asking for forgiveness because if reprehended, he might be incapable of holding himself back and could attack again.

His behavioral changes started after a serious accident at the age of nine. Close to his house there was a saw-mill, where workers used an iron bar to stop the pulley rotation after the machines were turned off. One day, he stuck the iron bar between the pulley arches to lock it while the machines were still on. The iron bar then span back striking him in the head. The trauma caused skull fracture and exposure of cerebral tissue. He did not lose consciousness and was seen by a doctor, who cleaned the wound and applied bandages.

After the accident his behavior changed radically. Before the accident he was a well-mannered boy at home and school, and an excellent student. But after the accident he became easily distracted, disobedient towards adults and teachers, and quarrelsome. As he grew older, his behavior deteriorated: he strived to be the center of attention, was loud and made inappropriate comments.

At the age of 18, he underwent neurosurgery to remove scar tissue, which worsened the severity of the symptoms but led to no additional symptoms. Even after getting married, he went out with other women and prostitutes, telling them he was a widower, and gave these women his home phone number.

Despite the behavioral changes, his cognitive performance seemed to be preserved. He was able to memorize long excerpts from the bible, was knowledgeable about his hometown's politics, and was occasionally hired to survey rural properties.

Despite his qualifications, he only managed to hold down menial jobs. He was employed at a public office only due to tolerance from his bosses and to his family's intervention.

During the interview and examination, he behaved well, but in the waiting room he talked loudly, tried to strike up conversation with other patients and exhibited puerile jocosity. His physical and neurological examination revealed no abnormalities, except for testing positive for Myerson's sign. He scored 30 on the mini-mental state examination.

**Figure 1:**
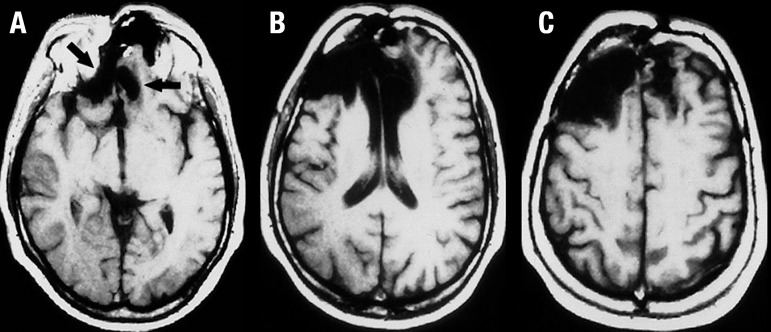
Axial brain MRI images. Axial T1-weighted images [A-C] demonstrate bilateral hypointense lesions in the ventromedial prefrontal cortex that are more extensive to the right side, where lesion also encompasses the adjacent orbitofrontal cortex [arrows in A]. There is also bilateral involvement of the anterior cingulate cortex and the genu of the corpus callosum [B]. The left lateral portion of the prefrontal cortex is spared; but to the right there is extension of the hypointensity especially to the dorsolateral cortex [these aspects are more evident in B and C].

**Figure 2:**
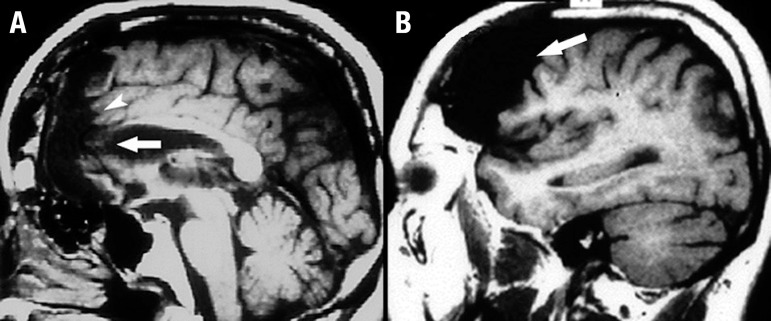
Sagittal brain MRI images. Sagittal T1-weighted images [A and B] show hypointense lesions in the genu of the corpus callosum [arrow in A] and anterior cingulate cortex [arrowhead in A]. The damage to the right medial prefrontal cortex [A] and the adjacent dorsolateral prefrontal cortex [arrow in B] is more clearly visible, while the ventrolateral cortex is partially preserved near the sylvian fissure.

**Figure 3:**
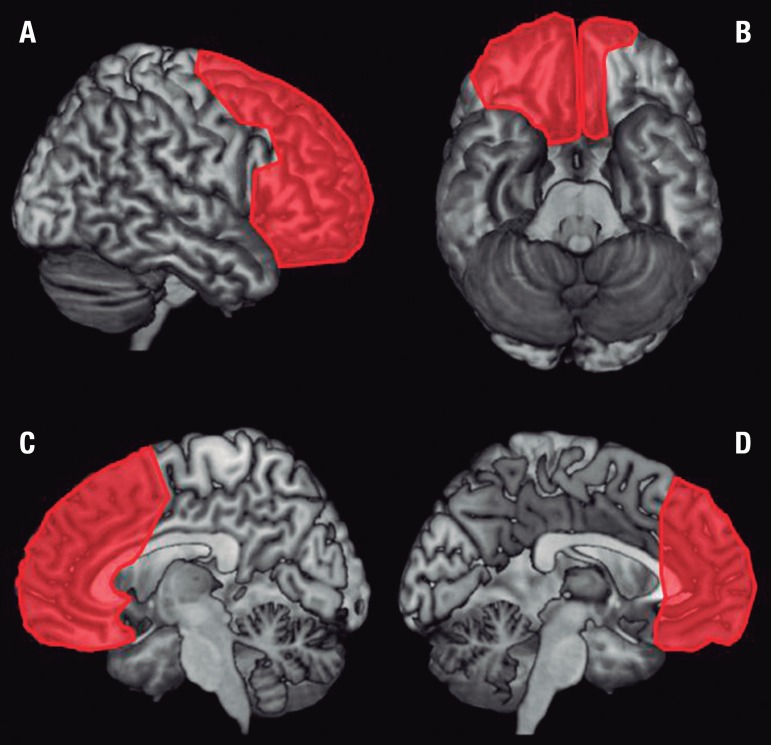
Representation of lesions on 3D brain model. [A] Right lateral view; [B] Inferior view; [C] Medial view (Right hemisphere); [D] Medial view (Left hemisphere). 3D template from MRIcron (Rorden, C., Brett, M. Stereotaxic display of brain lesions. Behavioural Neurology 2000;12:191-200.)

The electroencephalogram showed no abnormal activity. His brain MRI scan revealed bilateral orbitofrontal (OFC) and ventromedial prefrontal lesions that were more extensive to the right side (Figures 1, 2 and 3). The anterior cingulate cortex was also damaged bilaterally, as well as the genu of the *corpus callosum*. The right dorsolateral prefrontal cortex (PFC) was extensively damaged, while the ventrolateral PFC seemed to be spared. These two regions were spared on the left side.

The patient was treated with carbamazepine and pericyazine and advised to seek psychiatric treatment in his hometown. He showed some improvement but complained of excessive sleepiness.

He underwent neuropsychological assessment in 2000. During the evaluation, he was mildly agitated, verbose, and often had to be redirected to focus on the testing. His verbal IQ was 116, performance IQ 100, with a total IQ of 109 (WAIS). He had a normal performance on the following tests: go-no go tests, Hooper visual test and Raven progressive matrices, block design and Rey Complex Figure copy, attention tests, Wisconsin card sorting Test, arithmetic and similarity tests (WAIS), phonemic and semantic verbal fluency tests, Boston naming test and Rey auditory verbal learning test. His performance for delayed recall of the logical memory was at the 85^th^ percentile. He showed mild impairment in trail-making-B, digit-symbol, picture arrangement, object assembly (WAIS), and the visual reproduction test (WMS-R).^3,4^ Overall, the neuropsychological assessment showed mild impairment in attention/executive functions and visual episodic memory.

## DISCUSSION

This patient developed behavioral and personality changes, with great impulsivity, social disinhibition and poor job performance as a result of a frontal brain trauma during his childhood. Despite these symptoms, he had good cognitive performance, especially in activities of daily living that involved verbal memory.

In 1948, Ackerly and Benton (cited by Eslinger et al.)^5^ were the first to report a patient with a very early prefrontal lesion and characterized the neurodevelopmental abnormalities as a "primary social defect". Changes in social conduct with relative preservation of cognitive abilities have been described in patients with early frontal lesions. There is evidence that prefrontal lesions sustained during the perinatal and infancy periods have devastating consequences on the development of social behavior, personality and moral conduct.^2,5^ Such early deficits can become apparent only later in life, as it is believed that a certain degree of brain maturation and higher social demand are required for full expression of symptoms.^6^

In the majority of reported cases, the frontal lobe lesion occurred in children aged four years or younger.^7-9^ In these reports, lesions in the dorsolateral prefrontal region were associated with executive dysfunction and were less incapacitating than lesions in orbitofrontal, prefrontal ventromedial and polar frontal regions, which interfered in the correct development of social cognition with resultant impairments in inhibitory control, decision-making, moral judgment and empathy (theory of mind).^7^

Eslinger et al.^8,10^ reported two patients who suffered lesions later in childhood (at age 7) and highlighted that behavioral and personality changes were less severe than those observed in lesions occurring earlier in life.

The OFC receives afferent connections from the amygdala, cingulate gyrus, parahippocampal cortex and hippocampus and therefore receives sensory, emotional and memory-related information. Bilateral orbitofrontal damage leads to perseverative responses to previously rewarding stimuli, and deficits in decision-making.^11-13^ This can explain the present patient's impulsivity and inappropriate behavior, as he would have difficulty adapting his behavior to external stimuli. His impairment in inhibitory control shown by neuropsychological testing could also be similarly explained. The OFC, together with the anterior cingulate cortex, prefrontal ventromedial and dorsolateral regions, among others, is involved with empathy.^11-13^

The PFC ventromedial region is connected to many sensory modalities, the temporal lobe, insular cortex, as well as to the premotor cortex and basal ganglia, hence influencing the behavioral response. This region, together with the OFC, has also been associated with decision-making and emotion regulation.^14-16^

The PFC dorsolateral cortex has extensive connections with the temporal, parietal and unimodal visual cortices, amygdala and cingulate gyrus. This region is also implicated in reversal learning and attentional set maintenance, and thus in decision-making.^15^ Clinically, individuals with prefrontal dorsolateral lesions present with executive dysfunction.^17^ Lesion in the prefrontal dorsolateral cortex can therefore explain the deficit in attention and mild executive dysfunction evidenced by our patient's neuropsychological assessment.

The interesting aspect of this patient's symptoms is that many functions associated with the PFC and its connections were preserved despite the extensive lesion disclosed on the brain MRI. The fact that the patient felt guilty for his actions and had partial insight of his behavior is particularly striking. The feeling of guilt, together with pity and embarrassment, are considered prosocial sentiments that enable us to care about others and be aware of our mistakes.^18^ Prosocial sentiments are essential for moral conscience. In a functional neuroimaging study, Moll et al.^19^ suggested a critical role of the frontopolar cortex and septal region in enabling prosocial sentiments.

There are at least two distinctive facets of moral processing to consider: [1] an implicit and automatic level mediated through orbital and inferior mesial prefrontal regions, which is more emotionally based; and [2] a slower acting, cognitive level that contributes to moral reasoning and becomes alerted secondarily and is mediated through frontal polar and dorsolateral regions.^10,20-22^

In our patient, his inability to control impulses despite feeling guilt suggests that the first level of moral processing was much more disturbed than moral reasoning.

His clinical presentation has features also observed in emotionally unstable (borderline) personality disorder, which is usually associated with psychological trauma, severe neglect during childhood or severe parental inadequacy.^23^ Patients with borderline personality disorder may exhibit neuropsychological deficits.^24^

It is possible that the extent of the lesion, with less severe involvement of the left hemisphere, in which the left dorsolateral and ventrolateral PFC were not impaired, may be responsible for this relative preservation of moral processing. The right ventrolateral PFC was at least partially undamaged, another feature that may have contributed to the preservation of moral processing in this case. The age at which the lesion occurred could also have influenced his clinical presentation. At the age of nine years, many aspects of executive functioning, social cognition and self-regulation had already been developed, and most of the social rules had already been learned.^10^
